# Estren prevents beta-amyloid-induced basal forebrain cholinergic loss and long-term spatial memory deficits in aged female mice

**DOI:** 10.1038/s41598-026-49638-1

**Published:** 2026-04-27

**Authors:** John McLoughlin, Jessica Sayfullaeva, Elifnur Kiliç, Caolainn Carmody, Natalia Grochowska, Kyoko Potapov, Daniil Potapov, Andrew Clarkson, Katie Peppercorn, Warren Tate, Andrea Kwakowsky

**Affiliations:** 1 Pharmacology and Therapeutics, School of Pharmacy and Medical Sciences, Institute for Health Discovery and Innovation, Institute for Clinical Trials, Galway Neuroscience Centre, University of Galway, Galway, Ireland; 2 Department of Physiology, Faculty of Biomedical Sciences, University of Otago, Dunedin, New Zealand; 3 Department of Anatomy, Faculty of Biomedical Sciences, University of Otago, Dunedin, New Zealand; 4 Department of Biochemistry, Faculty of Biomedical Sciences, University of Otago, Dunedin, New Zealand; 5 Department of Pathology and Molecular Medicine, Faculty of Medicine, Dunedin, University of Otago, Dunedin, New Zealand

**Keywords:** Alzheimer’s disease, Basal forebrain cholinergic neurons, Estren, Estradiol, Amyloid beta, Neuroscience, Physiology

## Abstract

Basal forebrain cholinergic (BFC) deficits induced by amyloid beta (Aβ) are a well-established feature of Alzheimer’s disease (AD), and are linked to substantial cognitive decline. The gonadal steroid 17β-estradiol (E2) is neuroprotective; however, its therapeutic use is limited by adverse side effects. In contrast, activating non-classical E2 actions on intracellular signaling pathways offers therapeutic potential without invoking estrogenic off-target effects. Our previous studies demonstrated that after a highly toxic Aβ_1–42_ injection into the mouse basal forebrain, a single dose of 4-estren-3α, 17β-diol (estren), a selective activator of the non-classical estradiol pathway, prevented the loss of cholinergic signaling and also attenuated the Aβ_1–42_-induced learning deficits in young female mice. AD is an age-related disorder, and the most common cause of dementia in older adults. Therefore, in the current study, we examined the therapeutic potential of estren in aged (18-month-old) female mice. We found that estren prevented the Aβ_1–42_-induced loss of BFC neurons and the long-term spatial memory deficit following injection into the mouse basal forebrain of aged female mice. Notably, estren was more effective in reducing BFC cell death in aged mice following injection of Aβ_1–42_. The Aβ_1–42_ did not reduce the density of acetylcholine esterase-positive BFC projections in the somatosensory cortex of aged mice, as had been observed in young mice. Together, these findings suggest that selective activators of non-classical intracellular estrogen signaling when administered to older adults have the potential to reduce Aβ_1–42_-induced cholinergic neurodegeneration induced by increasing concentrations of Aβ and to preserve cognitive performance in AD.

**Supplementary Information:**

The online version contains supplementary material available at 10.1038/s41598-026-49638-1.

## Introduction

Alzheimer’s Disease (AD) is a progressive neurodegenerative disorder, which is characterized by global cognitive impairment, affecting memory, language and behaviour^1^. Two pathological hallmarks of AD are intracellular neurofibrillary tangles composed of hyperphosphorylated tau protein and extracellular aggregates of amyloid-beta (Aβ)^2^. Up to two-thirds of individuals affected by AD are women^3^, a disparity that may be partially explained by sex-specific hormonal differences between men and women. Estrogens, the primary female sex hormones, have been shown to exhibit neuroprotective effects^4^. Among these, 17β-Estradiol (E2), is the most potent and abundant form of these estrogens and exerts its effects through binding estrogen receptors (ERs) and stimulating both classical and non-classical signaling pathways^5,6^. The classical pathway involves the activation of the estrogen response element (ERE) and direct transcriptional activity, whereas the non-classical pathway activates gene transcription factors and functions independently of the ERE binding^6,7^. Both pathways are active in the brain, where estrogens regulate neuroprotection against inflammation and injury, spino- and synaptogenesis, synaptic plasticity, and memory function^6,8,9^. For this reason, estrogen-based hormone replacement therapy (HRT), has been investigated in large clinical studies such as Women’s Health Initiative (WHI) and Women’s Health Initiative Memory Study (WHIMS), to assess the impact of HRT on diseases such as breast cancer, cardiovascular disease, and dementia^10,11^. However, these studies identified several adverse effects, including an increased risk of breast cancer, stroke, pulmonary embolism, and myocardial infarction^10,12^. The validity of these studies has since been questioned due to the advanced age of participants at the treatment initiation^13^. Subsequent trials, including the Kronos Early Estrogen Prevention Study (KEEPS) and the Early versus Late Intervention Trial with estradiol (ELITE), provided evidence supporting the ‘critical window’ hypothesis, demonstrating beneficial cardiovascular outcomes of HRT when initiated within 10 years of menopause^13–15^.

Despite these findings, the impact of HRT on AD risk remains unclear due to the wide variety of HRT formulations, doses, and routes of administration, and interactions between genetics, environmental factors, and socio-economic factors^16^. Therefore, exploring the effects of alternatives to HRT may be beneficial for those with AD. These alternatives include selective estrogen receptor modulators (SERMs) and activators of non-genomic estrogen-like signaling (ANGELs)^17,18^.

The effects of SERMs are tissue-specific, that is they can exert both estrogenic and anti-estrogenic effects depending on the tissues targeted, for example, being an agonist in bone tissue but an antagonist in breast tissue^18,19^. Several SERMs, such as tamoxifen, raloxifene and lasofoxifene, are currently being utilized in the treatment of breast cancer, osteoporosis and postmenopausal symptoms^18,19^. The Multiple Outcomes of Raloxifene Evaluation Trial (MORET) showed that there was no significant difference in women taking a standard dose of raloxifene (60 mg/day) compared to a placebo in their risk of developing cognitive impairment, AD or other forms of dementia^20^. However, an increased dose of 120 mg/day showed a significantly decreased risk of mild cognitive impairment and a non-significant decrease in AD^20^. Some adverse effects have been identified with SERMs, including vasomotor symptoms, such as hot flushes, venous thromboembolisms and retinopathy^21^. A number of more recent studies have examined the effects of SERMs, including in relation to AD. In 2023, Branigan et al. completed a study to establish the risks estrogen-modulating therapies, comprising SERMs, steroidal aromatase inhibitors and non-steroidal aromatase inhibitors, have on neurological degenerative diseases (NDDs) such as AD, non-AD dementias, multiple sclerosis, Parkinson’s disease and amyotrophic lateral sclerosis^22^. They showed that estrogen-modulating therapies decreased the risk of NDDs by 15%, with tamoxifen decreasing it by 56% and the aromatase inhibitors decreasing it by 17%. Raloxifene, however, was found to increase the risk of NDDs by 18%^22^. The authors concluded that this was because estrogen-modulating therapies acted as partial estrogen agonists in neuronal tissues, as all therapies tested, except raloxifene, crossed the blood–brain barrier, and therefore reduced the risk of NDDs^22^. Cai et al. in 2024 also determined through a cohort study that hormone-modulating therapies used in breast cancer treatments, including SERMs, aromatase inhibitors and selective estrogen receptor degraders, were associated with an overall 7% decreased risk of AD and other related dementias^23^. The risk varied between age and race groups, with black women between 65 and 74 having a decreased risk of 24%, while white women in the same age cohort had a decreased risk of 11%. In black women 75 years and older, the risk decreased by 19%, while in white women, it decreased by only 4%^23^. Therefore, while these studies are promising, further in-depth studies are needed to support the use of SERMs to corroborate the findings.

ANGELs are a group of three compounds, estren, compound A and compound B, which selectively activate the non-classical E2 signaling pathway, but are not currently used clinically^6^. Preclinical studies indicate that these compounds may have a neuroprotective effect through the restoration of cholinergic fibre density in the somatosensory cortex following Aβ-induced injury of the basal forebrain^17,24^. In previous in vitro studies, estren has also been shown to exhibit both antiapoptotic and neuroprotective effects against Aβ toxicity^25,26^. With age as the primary risk factor for AD, studies conducted in aged animals are particularly valuable to understand disease pathology and responses to therapeutics. This present study aimed to evaluate the effects of estren on cholinergic dysfunction and long-term spatial memory following Aβ_1-42_-induced neurotoxicity, with a particular focus on the potential impact of aging on this process.

## Methods

### Animals

All experiments were approved and performed in accordance with the regulations of the Australian and New Zealand Council for the Care of Animals in Research and Teaching (ANZCCART) and the University of Otago (AEC No. 19/11 and AEC No. 60/13) and the University of Auckland Animal Ethics Committees (AEC No. 001655). All mice were bred and housed at the Hercus Taieri Resource Unit, University of Otago and Vernon Jansen Unit, University of Auckland. The animals were maintained under conditions of a 12-h light/dark cycle (lights on at 7:00 PM) with food and water available ad libitum except during behavioural experiments. All experiments were performed on aged adult (18-month-old, n = 14 per treatment group) C57BL/6 J wild-type strain female mice, and the Western blotting study also included a group of 21-month-old and 6-month-old female mice (n = 4 per group). All surgical procedures and behavioural experiments were performed as described in^24^. This current study and the earlier study performed using young mice^24^ were both carried out in the Behavioural Phenotyping Unit at the University of Otago using the same equipment and standardized procedures to minimize the impact of environmental bias and allow for direct comparison of the findings of the two studies.

### Aβ_1–42_ preparation

Aβ_1–42_ was produced as described in^24^ and^27^. The Aβ_1–42_ is routinely produced as a recombinant protein fused with maltose binding protein (MBP) with a proteolytic cleavage site for Factor X protease between the two segments^27^. This strategy utilises the solubilizing properties of MBP, the product of the *MalE* gene, to ensure expression of high concentrations of soluble protein in *Escherichia coli*. Following expression of the recombinant fusion protein in bacteria, the product was purified on an amylose column, to which the MBP segment binds. After binding to amylose resin, the pure fusion protein was eluted with maltose and concentrated by ammonium sulphate precipitation. The MBP carrier was subsequently cleaved off the fusion protein using Factor X protease, and the released Aβ_1–42_ peptide was isolated and further purified by hydrophobic chromatography with 0–50% v/v acetonitrile/0.1% v/v TFA, using Fast Protein Liquid Chromatography (FPLC). Fractions containing pure Aβ_1–42_ were identified immunologically with an antibody against residues 17–24 of Aβ_1–42_ and lyophilized to remove solvent. Mass spectrometry was then used to confirm the expected molecular ion for the desired product. Before intra-cerebral injection of the Aβ_1–42_, the prepared monomer was dissolved in artificial cerebrospinal fluid (ACSF: 147 mM Na + , 3.5 mM K + , 2 mM Ca2 + , 1 mM Mg2 + , pH 7.3)^24^ and^27^). The solution was ‘aged’ at room temperature (RT) for 48 h to facilitate the conjugation of toxic soluble aggregates, as documented by SDS/PAGE. The optimal incubation time for Aβ_1–42_ preparations to produce the highly toxic oligomers is 48–120 h^27^.

### In vivo experiments

**Fig. 1:**
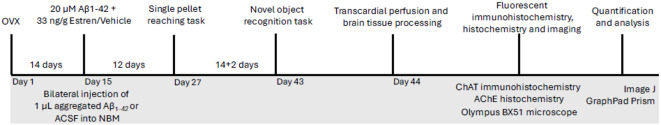
Experimental timeline and design.

The mice were anaesthetized with avertin (0.1 ml/10 g body weight) before bilateral ovariectomy (OVX) (Fig. 1). Two weeks after the OVX procedure that ensured fluctuating estrogen levels would not influence the results, mice were anaesthetized with isoflurane and given 1 µL aged Aβ_1–42_ diluted in ACSF slowly (0.1 μl/min) into the Nucleus Basalis of Meynert (NBM) bilaterally. Stereotaxically, the Aβ_1–42_ was injected relative to bregma at anteroposterior (0.7 mm), mediolateral (-2 mm), and dorsoventral (-3.75 and -4.75 mm, 0.5 μl at both coordinates) from dura^24^. Control injections were performed using 1 μl ACSF. Based on a previously established protocol, a 20 μM Aβ_1–42_ dose and a 12-day post-injection period were chosen for the subsequent behavioral experiments^24^. Estren treatment (estren 33 ng/g (Straloids) or vehicle (Ethyl oleate, an excellent solvent for steroids with low viscosity, absorption properties and providing high stability) was administered subcutaneously one hour after the Aβ_1–42_ administration^24^ (Fig. 1).

### Behavioral experiments and analysis

Twelve days after bilateral injection of Aβ_1–42_, the following behavioral tests were carried out (Fig. 1) as described in^24^. The single pellet skilled reaching task (performed on D27-D41) was carried out utilizing a three-lane plexiglas reaching apparatus (30 cm deep, 10 cm wide, and 30 cm high for each lane) to allow simultaneous recording of three animals^28^. Mice were fasted to 90% of their baseline body weight and maintained at this level throughout the 2-week testing period. On the first day, animals were habituated by placing them into the lanes for 15 min. On the following day, sugar pellets (20 mg, Bio-Serv) were freely available on the lane floor within tongue reach, as well as just outside the slot opening. Pellets were gradually removed from the floor until only the pellets positioned just outside the slots remained. Pellets were then gradually moved further away from the slot (up to a maximum distance of approximately 1 cm) to force the mice to use their paw rather than their tongue to reach for pellets. All mice were weighed daily and fed approximately 2 g of food after each training session to maintain their body weight at 90%. From day 2, pellets were presented one at a time, and reaching attempts were recorded with a video recorder. Each animal received a total of 15 pellets for each 15 min test period for a duration of 14 days (Fig. 1). The mice were scored for every reach if the mouse successfully brought the pellet back to its mouth and consumed it^28^. The single pellet reaching task was conducted during the middle of the dark cycle when the animal’s motivation to eat is very high.

The novel object recognition (NOR) test was conducted after a 2-day rest period after completion of the single pellet skilled reaching task (Day 43–44), to evaluate long-term recognition memory (Fig. 1). During the rest and testing period, animals had access to food and water ad libitum*.* The task was initiated at the start of the dark cycle (3 h into the dark cycle) when the activity levels of the mice are high. First, each mouse was placed in the arena individually and given 10 min to habituate to the environment. Next, animals were allowed to explore a set of two identical objects for a 5 min period, after which the mice returned to their cages. Twenty-four hours later, the animals were reintroduced to the same environment and presented with a similar set of objects, where one object was novel. The mice were allowed to freely explore the objects again for a 5 min period. The discrimination preference index, for a novel over a familiar object was calculated as follows: time near novel object less than the time near the old object, divided by time near the new object plus time near the old object^29^. Near the object or interaction time was defined as the time the animal had direct contact with the object, which includes any contact with the mouth, nose, paw only, or sniffing the object. The measurements were taken using manual stopwatches 3 times for each animal and averaged. No minimum exploration time was applied, all data were included in the analysis. No significant differences were found in the distance travelled between the different experimental groups. For these measurements, the centre-point of the mouse was detected and tracked, and the size of the behavioural arena was calibrated in the software to convert the animal’s motion from pixel shifts to distance moved. Animal behaviour was monitored, and the videos were analyzed using TopScan (CleverSys. Inc., USA) system.

### Tissue preparation

Following the behavioral testing (Day 44), mice were deeply anaesthetized with avertin, then perfused transcardially with 20 ml of ice-cold 4% paraformaldehyde in phosphate buffer (pH 7.6). Brains were extracted and post-fixed in paraformaldehyde solution for 2 h at 21 °C, followed by overnight incubation in 30% sucrose prepared in Tris-buffered saline (TBS) at 4 °C. Four series of 30-μm-thick coronal brain sections were cut using a freezing microtome. The sections were then stored in an antifreeze solution (5.72 mM NaH_2_PO_4_, 38.2 mM Na_2_HPO_4_, 20% glycerin, and 30% ethylene glycol) until use^24^.

### Immunohistochemistry

Free-floating peroxidase-based immunohistochemistry for choline acetyltransferase (ChAT) was undertaken as per previously published studies^24,30^. One series of brain sections was incubated with primary antibodies recognizing ChAT (, Table 1). This was followed by incubation of sections with a biotinylated donkey anti-goat IgGs, (Table 2) and the avidin–biotin-HRP complex (1:200; Vector Elite ABC kit, Vector Laboratories). Labeling was then visualized with nickel-diaminobenzidine tetrahydrochloride (DAB) using glucose oxidase, resulting in a black precipitate within the labeled cells^24^.

### Acetylcholine esterase (AChE) histochemistry

AChE histochemistry with silver nitrate intensification was performed to label and visualize cholinergic fibres in the cortex^24,30,31^. One series of brain sections was incubated in a buffered solution of sodium acetate (0.1 M; pH 6), acetylthiocholine-iodide (0.05%), sodium citrate (0.1 M), copper sulfate (0.03 M), and potassium ferricyanide (5 mM). This was followed by incubations with ammonium sulfide (1%) and then silver nitrate (1%)^24^.

**Table 1:** Primary antibodies used in this study.

Antigen	Immunogen	Source, HostSpecies, Catalogue Number	Dilution for immunohistochemistry	Dilution for Western blotting
ChAT	Human placental enzyme	Millipore (Chemicon), AB144, RRID: AB_90650	1:2000	NA
ERα	Synthetic peptide within human estrogen receptor alpha	Li-Cor, Goat, 926-32210, RRID: AB_3096013	NA	1:10,000
β-actin	Synthetic peptide corresponding to beta actin amino acid 1–14 (N terminal) conjugated to Keyhole Limpet Haemocyanin	Li-Cor, Goat, 926-32211, RRID: AB_621843	NA	1:10,000

**Table 2:** Secondary antibodies used in this study.

Antigen	Immunogen	Source, HostSpecies, Catalogue Number	Dilution for immunohistochemistry	Dilution for Western blotting
Biotin SP Donkey anti-Goat IgG	Polyclonal Gamma Immunoglobins Heavy and Light chains	Jackson, Donkey, 705,065,147, RRID: AB_2340397	1:200	NA
Goat Anti-Mouse IgG	Polyclonal Gamma Immunoglobins Heavy and Light chains	Li-Cor, Goat, 926–32,210, RRID: AB_3096013	NA	1:10,000
Goat Anti-Rabbit IgG	Polyclonal Gamma Immunoglobins Heavy and Light chains	Li-Cor, Goat, 926–32,211, RRID: AB_621843	NA	1:10,000

### Analysis of histological data

Cholinergic cell body and fibre loss data were analyzed as described^24^. The Paxinos and Franklin (2001) brain atlas was used to count ChAT-positive cell bodies in the NBM on both sides of the brain and the medial septum (MS). Three sections starting from the bregma – 1.2, with 120 μm inter-sectional distance from each animal was selected and analyzed for ChAT cell counting in the NBM, seven sections starting from the bregma + 1.18, with 120 μm inter-sectional distance from each animal, were selected and analyzed for ChAT cell counting in the MS, and ten cortical sections from each animal were chosen and analyzed for the AChE fibre density measurements (plates 28–40). ChAT cell counts were quantified as total cell numbers, and fibre density was normalized to the naive control group. An Olympus BX51 microscope was used to examine sections with ChAT and AChE labeling. Cell P-Image Analysis software was used to measure the density of cortical AChE-positive fibres. All measurements were conducted by an investigator blinded to the experimental groups. All cell counting and density measurements were performed independently by two investigators, and the data were averaged.

### Western blotting

Mice were euthanized by cervical dislocation, and the brain was removed, and the medial-septal diagonal band of Broca, including the NBM and MS, was dissected on ice. The brain samples were then freshly snap-frozen on dry ice and stored at -80 °C. Brain samples from each mouse were numbered at the time of the tissue collection, and the experimenter performing the Western blotting and analysis was blinded to the experimental grouping. Brain samples (n = 4 young female and n = 4 old female) were retrieved from the -80 °C freezer and immediately placed on ice. Brain tissue was lysed by adding 300-400µL (based on tissue size) of RIPA Lysis Buffer supplemented with a protease inhibitor cocktail (P8340, Sigma-Aldrich). Samples were homogenized using the sonicator (Brandon, Sonifier 150) for 10 s at 5.0 intensity to release proteins by bursting the cell membrane. Samples were then incubated on a rocker (Stuart, SSL4) for 45 min at 4 °C, then centrifuged at 4 °C, 13.000 rpm for 20 min (Hettich, Mikro 220R), then immediately placed on ice. The supernatant was collected and stored at -20 °C. The protein concentration of each sample was determined using a Bradford protein assay as per the manufacturer’s instructions (23,200, Thermo Fisher Scientific).

Thirty µg of each protein extract was run on a 12% SDS-PAGE gel at 80 V for 20 min, then at 100 V for approximately 60 min. A molecular weight ladder (928,600,000, LI-COR) was also loaded in gels. The samples were transferred onto a nitrocellulose membrane (10,600,003, GF Healthcare) for 1 h at 100 V, and then stained with Ponceau-S to visualize the proteins and confirm successful transfer. Membranes were then washed in 1 × Tris-Buffered Saline pH 7.6, 0.1% Tween 20 (TBST) for 5 min. The membranes were blocked in 1% Bovine Serum Albumin (BSA; A8022, Sigma-Aldrich) -TBST for 30 min at 4 °C. Membranes were then incubated with primary antibodies, ERα and β-actin (Table 1) diluted in 1% BSA-TBST at 4 °C overnight.

The following day, the membranes were washed with TBST three times for 5 min each, and the secondary antibodies were added at a 1:10,000 dilution (Table 2) and incubated at RT for 1 h. The membranes were washed with TBST 3 × 10 min, then with 1X TBS for 10 min on a rocker. Membranes were imaged using LI-COR imaging system (OFC-1025, LI-COR, Biosciences). The analysis was undertaken using ImageJ software (1.53 k) to measure the signal intensities of each sample and was normalized to β-actin.

## Statistical analysis

Statistical analysis was carried out using GraphPad Prism 10.4.2. Both the behavioral and Western blotting data followed a normal distribution; therefore, one-way ANOVA with post hoc Tukey test was applied to all data, but a repeated measure and/or two-way ANOVA was used to analyze the single pellet reaching behavioural data. An unpaired two-tailed t-test was carried out on the Western blotting data. Data in all experiments are expressed as mean ± SEM, where results were deemed statistically significant with a value of p < 0.05. The experimenter was unblinded after the statistical analysis was completed. Photoshop CC 2025 (Adobe) was used to prepare the figures.

## Results

### Did Aβ_1-42_ and/or estren influence reaching performance on the single pellet retrieval task in aged mice?

**Fig. 2:**
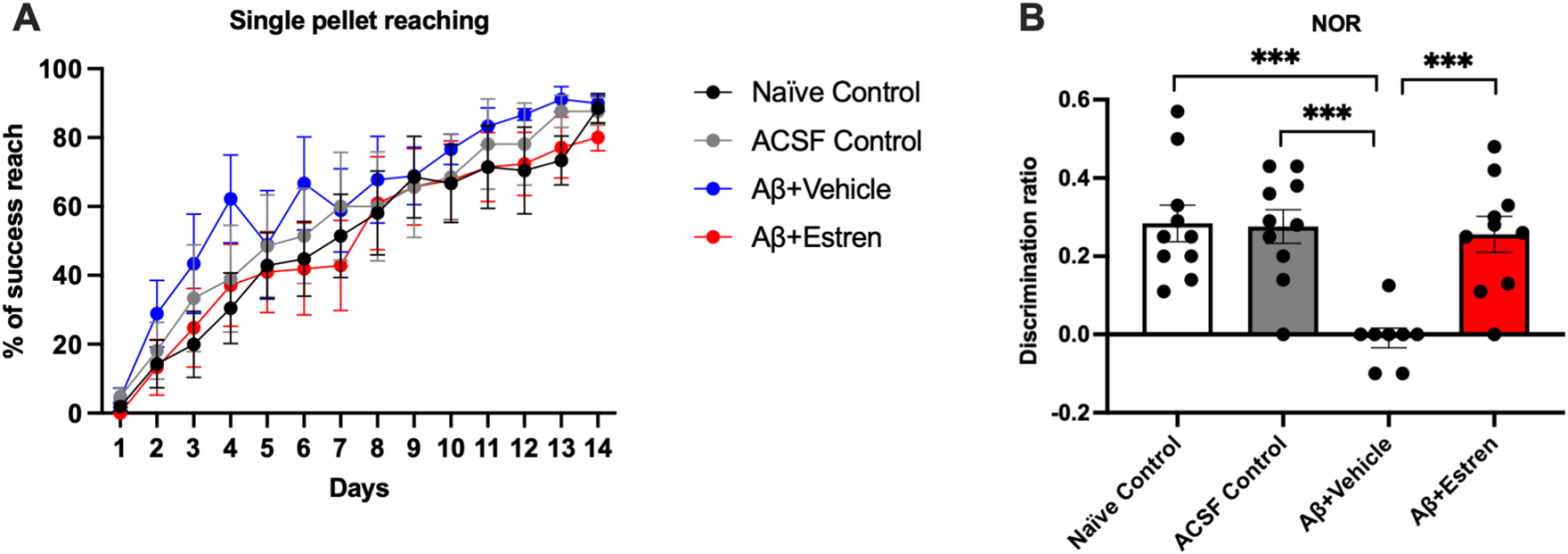
Estren restores long-term spatial memory deficits induced by Aβ_1-42_. A. Percentage of success reach in single pellet retrieval task. Aβ_1-42_ and estren did not influence reaching performance on the single pellet retrieval task. Values were converted to percentages (retrieval of 15 pellets = 100%). Histogram shows mean ± SEM (n = 14 per group). Repeated measure two-way ANOVA with Tukey’s post hoc. B. Estren treatment successfully rescued reaching performance on the novel object recognition (NOR) task in Aβ_1–42_– lesioned mice. Histogram shows mean ± SEM (n = 8–10 per group), ***p < 0.001. ANOVA with Tukey’s post hoc.

We first examined whether bilateral Aβ_1-42_ administration into the NBM induced a motor learning behavioral deficit and whether estren treatment influenced this outcome. Earlier studies demonstrated that disruption to the BFC system by Aβ_1-42_ impairs motor learning as assessed by the single pellet retrieval task in young female mice^24^. In contrast, in this study with aged female mice, Aβ_1-42_ did not influence the reaching performance on the single pellet retrieval task (Fig. 2A)*.* Similarly, estren also did not alter the performance of the mice in the pellet retrieval task compared to the control groups (Fig. 2A).

### Can estren restore long-term spatial memory deficit induced by Aβ_1-42_ in aged female mice?

While motor learning was not affected by Aβ_1–42_ administration, it did however result in a long-term spatial memory deficit in aged mice, as measured by the NOR task (naïve control vs Aβ_1–42_ + vehicle p = 0.0002; ACSF control vs Aβ_1–42_ + vehicle p = 0.0003) (Fig. 2B), consistent with the observations in young mice^24^. Importantly, it was encouraging to observe that estren significantly attenuated these deficits in the NOR discrimination index following Aβ_1–42_ administration (Aβ_1–42_ + vehicle vs Aβ_1–42_ + Estren p = 0.0009) (Fig. 2B).

### Estren treatment reduces cholinotoxicity in aged female mice

**Fig. 3:**
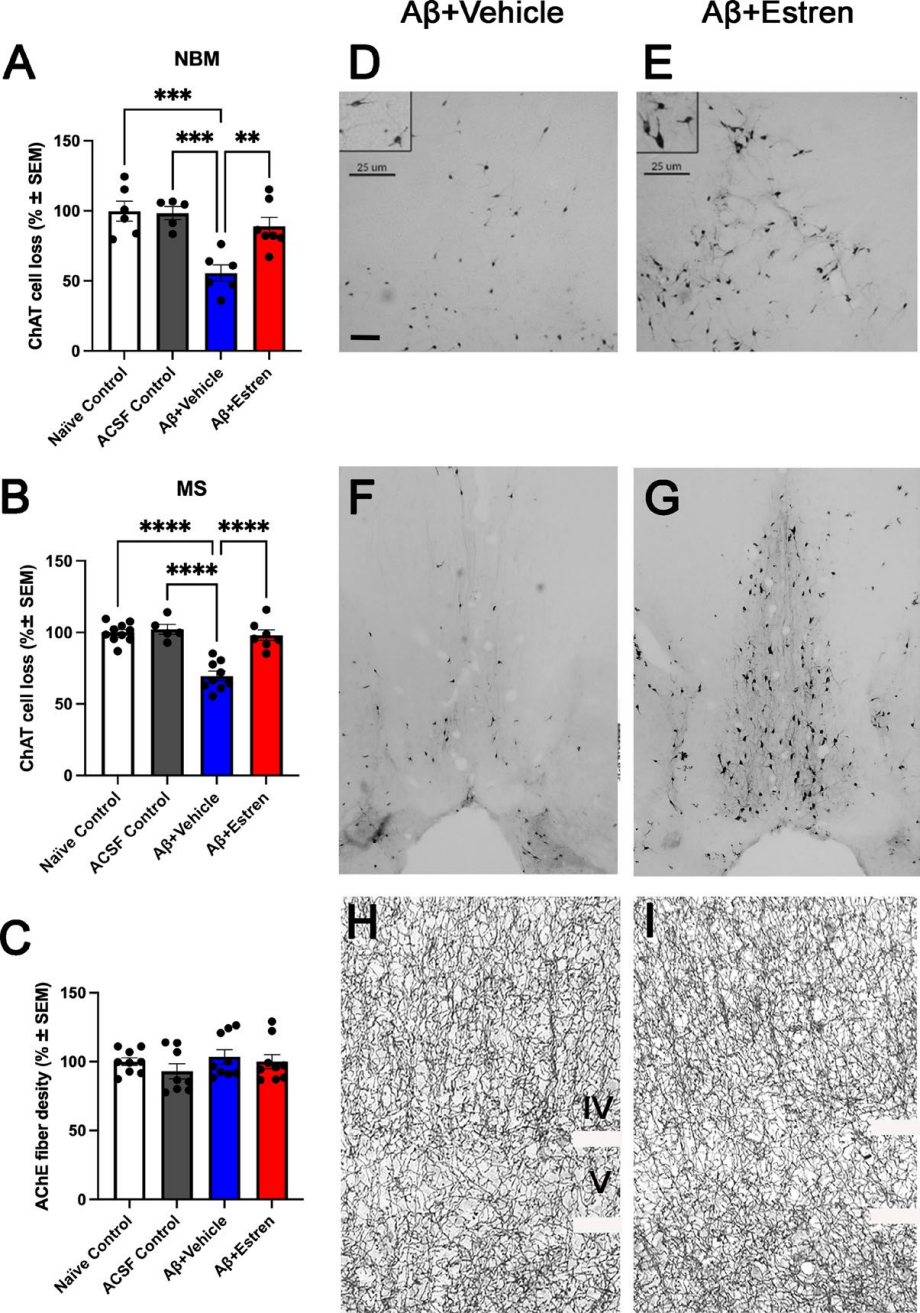
**A**, **B**. Estren treatment significantly reduces cholinotoxicity. Aβ_1-42_ significantly reduced cholinergic cell numbers in the NBM and MS compared to control groups and a single dose of estren restored cholinergic cell numbers. **C**. AChE fibre density is not affected by Aβ_1-42_ and estren treatment. Aβ_1-42_ and a single dose of estren do not significantly alter cholinergic fibre density in the somatosensory cortex compared to control groups (**C**, **H**, **I**). **A**-**C**. Histograms show mean ± SEM (n = 6 -11), **p < 0.01, ***p < 0.001, ****p < 0.0001. ANOVA with Tukey’s post hoc.

We next assessed whether Aβ_1-42_ induced cholinergic cell and fibre loss and if estren treatment ameliorated these effects, using ChAT immunohistochemistry and AChE histochemistry (Fig. 3). The observations from behavioural experiments were supported by the morphological data since estren treatment significantly reduced cholinotoxicity in the NBM (p = 0.0045) (Fig. 3A,D,E) and MS (p < 0.0001) (Fig. 3B,F,G) after bilateral Aβ_1–42_ administration. In contrast, Aβ_1–42_ did not decrease the AChE- positive cholinergic fiber density in somatosensory cortex in aged mice, and neither did estren alter the fiber density (Fig. 3C,H,I).

### Age does not affect ERα expression in the basal forebrain of female mice

**Fig. 4:**
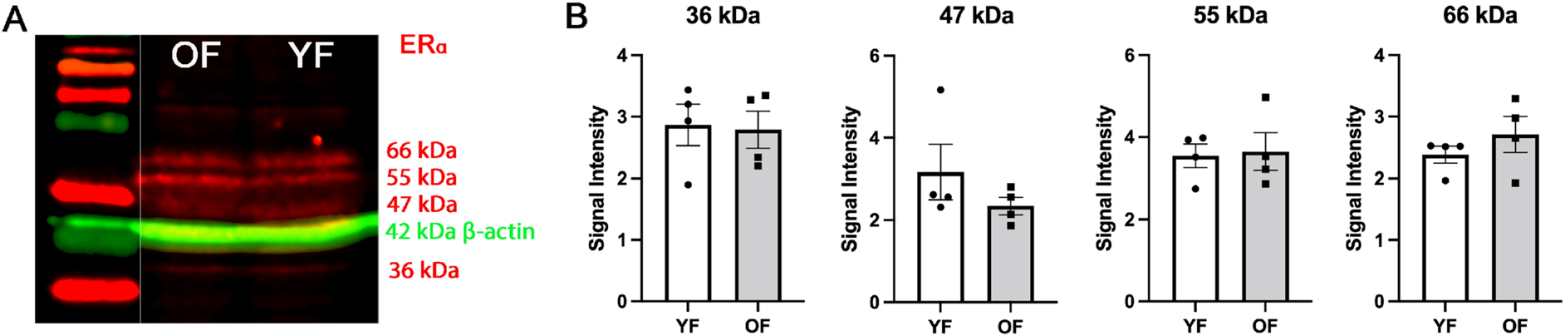
Representative immunoreactive Western blot bands from young female (FY) and old female (FO) basal forebrain homogenates following incubation with antibodies to the ERα and β-actin (**A**) and corresponding signal intensity graphs (**B**). A: Molecular markers (ladder) are shown on the left. The molecular weights of β-actin and ERα splice variants are shown on the right. B: Signal intensity for each ERα splice variant Western blot band was measured and normalized to their corresponding β-actin signal for each age group. The data are graphed as ± SEM (n = 4; unpaired two-tailed t-test).

In the following experiments, we examined whether ERα expression in the basal forebrain was comparable between young and old female mice. Previous studies have demonstrated that E2 and estren actions rely on neuronal ERα for neuroprotection on cholinergic neurons in the basal forebrain of young mice following Aβ_1–42_- and N-methyl-D-aspartate (NMDA)-induced neurotoxicity^24,30^. The results of this study show no significant difference in the protein level of ERα in the basal forebrain between young and aged female mice (Fig. 4A,B).

## Discussion

We report here that a single dose of estren treatment ameliorates Aβ_1–42_-induced cholinergic cell loss in the NBM and MS of aged female mice, and it effectively prevents the development of recognition memory deficits. Notably, Aβ_1–42_ lesioning to the NBM did not induce AChE fiber density loss in the somatosensory cortex or motor learning deficits, as we had earlier observed in young female mice.

In the pathogenesis of AD, there is an increased concentration of toxic soluble Aβ oligomers and cholinergic cell loss^32^. The cholinergic neurons of the NBM projecting to the somatosensory cortex are particularly sensitive to Aβ_1–42_-induced toxicity^33^. The loss of memory in AD has been associated with impaired cholinergic function in the basal forebrain, where declining activity of cholinergic neurons disrupts their projections to the cerebral cortex and hippocampus^33^. The available transgenic animal models of AD do not effectively model the sporadic forms of AD and the cholinergic deficit accompanying the disease^34–36^. With established research on the soluble Aβ oligomer-induced cholinotoxicity, we injected Aβ_1–42_ intracerebrally into the NBM in our experiments. The results demonstrated that Aβ_1–42_ was capable of damaging 60% of the BFC neurons in the NBM and 60–80% in the MS, impairing long-term spatial memory. Other laboratories have also shown that injecting the toxic soluble oligomers of Aβ into the NBM results in cell loss within the NBM, cortical cholinergic fibre loss and concomitant memory deficits in young 3–4-month-old mice^37–39^.

In regard to estren exposure, previous in vivo mouse experiments had demonstrated that BFC neurons responded to estren treatment rapidly and directly through the MAPK/CREB signaling pathway^17,24^. Estren did not prevent cholinergic cell death, and a significant cholinergic cell loss was induced by Aβ in young female mice^24^. However, the treatment has been found to have sprouting effects on AChE fibres, which is dependent upon activation of intracellular signal transduction pathways, including MAPK and CREB pathways and ERα^24,40–44^. Notably, the cholinergic cells in the NBM of young mice are highly sensitive to Aβ_1-42,_ and, while estren was unable to prevent cell loss, most likely this was because of immediate cell death on exposure to the Aβ_1-42_ or irreversible damage to the cells by the time estren reached them.

Despite the similar cholinergic NBM cell loss in young and aged mice, after 30 days of Aβ_1–42_ administration, no significant loss in AChE fiber density is detected in the somatosensory cortex of the aged mice, whereas young mice showed reduced fiber density. Therefore, cholinergic neuronal fibers might regenerate more efficiently in aged mice. Alternatively, ChAT levels might decrease markedly immediately after Aβ_1–42_ administration while cholinergic cell bodies are still intact. However, because the degrading enzyme AChE is more stable than the synthetic enzyme ChAT in the AD brain^45^, fibers projecting to the somatosensory cortex of these neurons are still detectable, and the networks are functional. A 40–50% decrease in ChAT activity in several brain regions of the *Chat* + /- mice resulted in no difference in AChE activity when compared to wild-type mice^46^. These mice also show normal performance in several behavioral assays, suggesting compensatory mechanisms must be in place to maintain normal cholinergic transmission when ChAT activity significantly decreases^46^. Both scenarios might explain the lack of deficits detected in the single-pellet reaching task, which requires a functional cholinergic network in the somatosensory cortex.

However, the significant 60–80% loss of cholinergic cells in the MS most likely underlies the long-term spatial memory deficits observed in aged mice in the NOR, a hippocampus-dependent task. A limitation of the study is the lack of hippocampal fiber density data, but likely due to the significant cell loss in the MS, the sprouting of these fibers innervating the hippocampus might have been seriously compromised, leading to impaired cognitive performance on the NOR. Importantly, a single dose of estren treatment was able to prevent the cholinergic cell loss in the MS and NBM, as well as learning deficits in old female mice. In young mice, estren did not significantly affect cholinergic cell loss in the NBM; however, there was a trend towards increased cell numbers, suggesting a protective effect that did not reach statistical significance^24^. This indicates that estren is more effective in aged female mice compared to young mice. These age-related differences in estren’s action cannot be explained by differences in BFC cell numbers or the expression levels of the receptors mediating the effects of estren. Previous research reports that the number of cholinergic cell bodies in the NBM and density of cholinergic fibres in the cortex are neither age- nor sex-dependent^47^.

Furthermore, we report that age does not affect ERα expression in the basal forebrain, as young and old female mice did not exhibit significant differences in ERα expression. In neurons, estrogen-induced responses range from enhancing survival to preventing cell death and facilitating neurite outgrowth^43^. Mouse models have previously shown decreased neuronal responses to E2 during aging with E2-desensitization, with an association with reduced neuronal ERα^48^. In a study investigating the effects of estrogen on the ultrastructural distribution of ERα on young and aged mice, it was found that in aged mice (22–23-months), there was decreased responsiveness of hippocampal synapses to E2, which may have resulted from age-related 50% decrease in ERα-containing spines^49^. Therefore, while overall ERα levels do not change during aging in the basal forebrain, its altered spatial distribution and function can underlie altered response to E2 and estren. Another mechanism to consider is the possible activation of androgen receptors (ARs) by prolonged high-dose estren teratment^17^. While we did not study AR expression, previous studies extensively examined AR expression in aged subjects, and reported a decrease in the human NBM and basal forebrain^50^ and hippocampus^51^, and also in the mouse and rat cortical and mesocorticolimbic areas^52,53^, while in aged male rats an upregulation was detected in the hippocampus^54^. Therefore, it is possible that estren action in the aging mouse brain could differ from that in young mice due to altered AR expression and function.

Overall, E2 levels of normal female mice decrease with chronological and ovarian aging^55^. Ovarian hormone loss leads to hippocampal-dependent cognitive impairment and synaptic loss in mid-aged and old, but not in young female mice^56^, suggesting that molecular and cellular changes are required in addition to hormonal loss to impact cognitive performance. Some studies have shown that aged mice exhibit significant deficits in spatial memory compared to young mice^57^. Female mice aged five, 17 and 25 months were assessed in a variety of behavioral tasks, including the Morris Water Maze (spatial and non-spatial reference memory), simple odor discrimination (olfactory reference memory), plus maze (anxiety/exploration). In this study, relative to five-month-old mice, 25-month-old mice had impaired spatial and olfactory reference memory, but intact non-spatial reference memory, and the spatial reference memory of 17-month-old mice was also impaired, although less so than the older 25-month-old mice^58^. Estrous cycling in females significantly decreased with increasing age as all 25-month-old females had finished cycling, and 80% of the 17-month-old females displayed either irregular or absent estrous cycling^58^. This suggests that the disruption of the estrous cycle has a negative impact on spatial memory throughout the aging process^59^. Estrus cycling and circulating blood E2 levels were not measured in the current study, but all mice were retired breeders with less frequent and reduced litter size 3–4 months before the start of the experiment. In addition, we previously reported that estren lacks uterotrophic activity, demonstrating the absence of an E2-mediated proliferative effect via classical genomic E2 action^17^.

In OVX mice, ongoing E2 treatment improves memory, as assessed by the NOR, through physiological effects reported in the medial temporal lobe of young mice (8 weeks)^60^ and the cerebral cortex of middle-aged female mice^61^. Multiple studies have demonstrated that estrogen treatment can enhance hippocampus-dependent memory in young OVX mice (2-month and 3-months, respectively)^62–65^ relative to non OVX controls. In middle-aged OVX rodent models, estrogen interventions targeting hippocampal ERα reveal improvements in spatial memory and sustain ERE-dependent gene expression after treatment has been completed^66,67^.

However, as mentioned earlier, only specific aspects of memory are impacted by age, making it difficult to compare results across studies. Also, the housing conditions and general health of the animals have a significant impact on the results. In our study, the long-term spatial memory assessed by the NOR test in 19-month-old female mice was not impaired compared to that of young mice^24^. While severe cognitive decline is not a normal part of aging, subtle age-related cognitive changes could be expected, but this might not have yet occurred at the age of the mice we studied. While this is a limitation of the study, using older mice raises ethical concerns of health issues, as a significant number of mice are suffering from severe health conditions and are at risk of dying if the starting point of the study is above 20 months (~ 30–50%).

The idea of developing compounds that do not activate the classical genomic estrogen pathways led to the discovery of several therapeutics with the promise to develop drugs that can prevent or treat AD. The estren treatment we have utilized does not exert ERα-mediated classical genomic action on the uterus^17^. Estren, which restores BFC neurons via the ERα pathway, does not involve genomic processes^24,25,30,68^. The non-classical actions are suggested to be involved in the estren-induced restorative mechanisms in BFC neurons. While these ‘ANGELS’ might have great potential for treating AD, more research is required to assess their clinical safety and efficacy.

A SERM, STX, which binds a G protein-coupled estrogen receptor GqMER and not nuclear ERs, is neuroprotective against Aβ-induced cytotoxicity in neuroblastoma cells^69^. STX also increased dendritic arborisation and spine densities of hippocampal neurons, after an Aβ-induced reduction^69^. Estrogens also play a role in schizophrenia, with postmenopausal women displaying worse negative and cognitive symptoms than premenopausal women^70^. Patients also show decreased activity in the prefrontal cortex during inhibition of emotional responses, a particularly sensitive to hormones^71^. A number of studies have shown that, when treated with a SERM, raloxifene, patients with schizophrenia have improvements in symptom severity, cognition and brain activity, as well as improvements in negative symptoms in postmenopausal women^70–72^.

In addition to ANGELs and SERMs, Pathway Preferential Estrogens (PaPEs) have been developed that are structurally altered ligands with reduced binding affinity to ERs, and they preferentially activate the extranuclear-initiated signaling pathway^73^. In *in vitro* studies, using primary neocortical cell cultures, PaPE-1 significantly inhibited Aβ-induced LDH release, ROS production and mRNA expression of apoptosis-related genes and other proteins. This PaPE was also observed to partially reverse the Aβ-induced reduction in cellular viability and mitochondrial membrane potential through the selective activation of extranuclear ERs, suggesting a potential application in AD^74^. The research to discover an alternative therapy to E2 continues, and effective activators of the non-classical estradiol signaling pathway might offer a safe option.

## Conclusions

In summary, based on our results, it can be concluded that Aβ_1-42_ lesioning of the NBM diminishes long-term spatial memory in aged mice, as assessed by NOR, which is similar to the effects of Aβ_1-42_ in young mice. Estren is shown in NOR to preserve long-term spatial memory. Neither Aβ_1-42_ nor estren, however, affect the motor learning of mice in the single-pellet reaching test in aged female mice. Aβ_1-42_ causes significant ChAT neuronal cell death in the NBM and MS of aged mice. In both the NBM and MS, estren prevents this ChAT neuronal cell death. When estren was administered after Aβ_1-42_ lesioning in young mice, a trend toward increasing ChAT cell numbers was observed; however, this did not reach statistical significance. These results indicate that estren is a more effective treatment in aged mice. To develop future therapies for AD-related cholinergic loss, more work is needed to map estren’s activity and attempt to identify more effective activators of the non-classical estradiol signaling pathway.

## Supplementary Information

Supplementary Information 1.

## Data Availability

Data is available within the main manuscript and supplementary material.
