# Supplementary figures and images for: Estren prevents beta-amyloid-induced basal forebrain cholinergic loss and long-term spatial memory deficits in aged female mice
Source: Sci Rep. 2026 Apr 27;16:19310. doi: 10.1038/s41598-026-49638-1 (PMC13284400; doi:10.1038/s41598-026-49638-1)

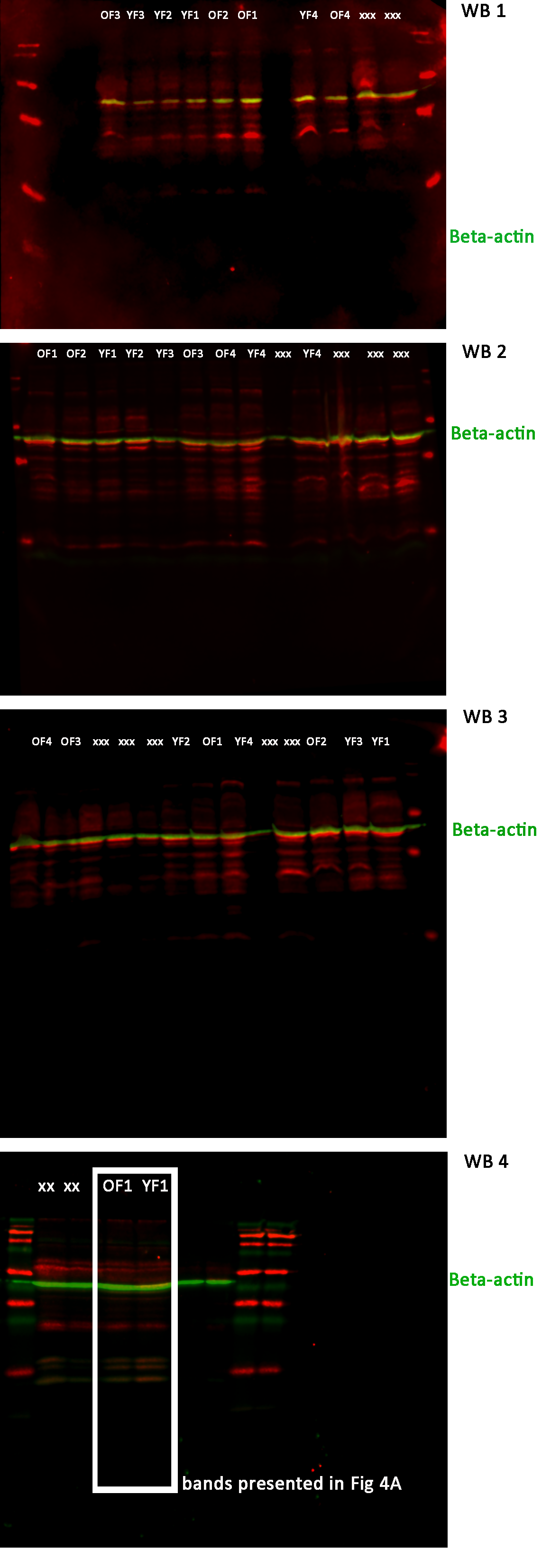

Supplement: Supplementary file 1 — Supplementary Information 1. [file 41598_2026_49638_MOESM1_ESM.tif]
